# MRI of the cervical spinal cord predicts respiratory dysfunction in ALS

**DOI:** 10.1038/s41598-018-19938-2

**Published:** 2018-01-29

**Authors:** G. Grolez, M. Kyheng, R. Lopes, C. Moreau, K. Timmerman, F. Auger, G. Kuchcinski, A. Duhamel, P. Jissendi-Tchofo, P. Besson, C. Laloux, M. Petrault, J. C. Devedjian, Thierry Pérez, Pierre François Pradat, L. Defebvre, R. Bordet, V. Danel-Brunaud, D. Devos

**Affiliations:** 1 Service de Neurologie, Université de Lille, CHU de Lille, INSERM UMRS_1171, LICEND COEN Center, Lille, France; 2 Département de Biostastistiques, Université de Lille, CHU de Lille, Lille, France; 3 Service de Neuroradiologie, Université de Lille, CHU de Lille, INSERM UMRS_1171, LICEND COEN Center Lille, Lille, France; 4 Service de Pharmacologie, Médicale Université de Lille, CHU de Lille, INSERM UMRS_1171, LICEND COEN Center Lille, Lille, France; 5 Plateau d’imagerie préclinique, Université de Lille, CHU de Lille, Lille, France; 6 Department of Radiology, Neuroradiology section, Free University of Brussels, CHU Saint-Pierre, Brussels, Belgium; 7 Service de Pneumologie, Université de Lille, CHU de Lille, Lille, France; 8 Laboratoire d’Imagerie Biomédicale, CNRS, INSERM, Sorbonne Universités, UPMC Univ Paris 06, Paris, France; 9 Département de Neurologie, Centre référent SLA, APHP, Hôpital Pitié-Salpêtrière, Paris, France

## Abstract

For patients with amyotrophic lateral sclerosis (ALS), the primary therapeutic goal is to minimize morbidity. Non-invasive ventilation improves survival. We aim to assess whether Magnetic Resonance Imaging (MRI) of the cervical spinal cord predicts the progression of respiratory disorders in ALS. Brain and spinal MRI was repeatedly performed in the SOD1^G86R^ mouse model, in 40 patients and in healthy controls. Atrophy, iron overload, white matter diffusivity and neuronal loss were assessed. In Superoxide Dismutase-1 (SOD1) mice, iron accumulation appeared in the cervical spinal cord at symptom onset but disappeared with disease progression (after the onset of atrophy). In ALS patients, the volumes of the motor cortex and the medulla oblongata were already abnormally low at the time of diagnosis. Baseline diffusivity in the internal capsule was predictive of functional handicap. The decrease in cervical spinal cord volume from diagnosis to 3 months was predictive of the change in slow vital capacity at 12 months. MRI revealed marked abnormalities at the time of ALS diagnosis. Early atrophy of the cervical spinal cord may predict the progression of respiratory disorders, and so may be of value in patient care and as a primary endpoint in pilot neuroprotection studies.

## Introduction

Amyotrophic lateral sclerosis (ALS) is a devastating neurodegenerative disease characterized by loss of upper and lower motor neurons. The main objective in patient care is to minimize mortality and maximize quality of life^1^. Respiratory support (i.e. non-invasive ventilation) is associated with longer survival^1^. Biomarkers might allow earlier, more effective care. Many studies have highlighted severe alterations in motor cortical areas and corticospinal tracts^2–4^.

To assess the potential value of Magnetic Resonance Imaging (MRI) as a surrogate marker, we compared conventional clinical findings (including respiratory disorders) and analyses of (i) the cervical spinal cord and (ii) a novel iron accumulation parameter. We also analysed the relationship between atrophy and iron accumulation in the *Sod1*^G86R^ mouse model. Next, we performed a longitudinal MRI assessment of the brain and the spinal cord in early-stage limb-onset ALS patients (to avoid the bias associated with bulbar/cervical onset); several recommended sequences were used to measure atrophy, iron overload, white matter diffusivity, and neuronal loss.

## Results

### MRI findings in a murine model of ALS

**Figure 1:**
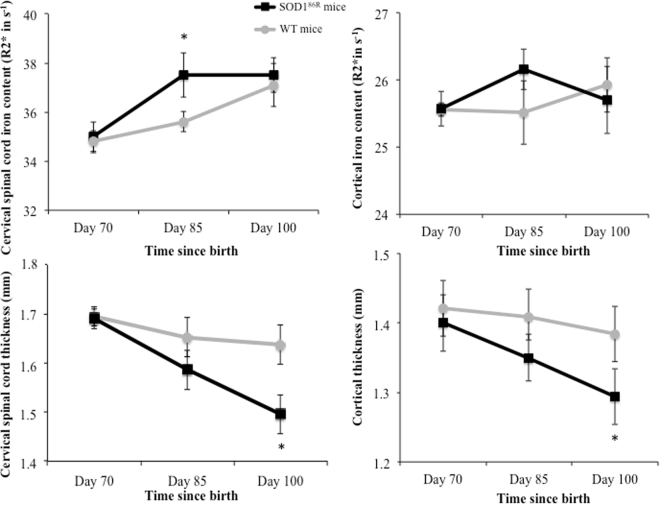
MRI findings in SOD186R mice. MRI findings in SOD1^86R^ mice versus wild type mice (WT). *Means p < 0.05 (ANCOVA adjusted on the baseline values (i.e. Day 70)).

No changes were observed at D70, i.e. an age at which Sod1^G86R^ mice are devoid of motor symptoms^5^ (Fig. 1). A markedly elevated R2* value in the cervical spinal cord was the first significant abnormality at D85, when the mice became symptomatic in the limbs (relative to controls)^5^. At D100 (shortly before death at D110), a significant decrease in the volumes of the cervical spinal cord and the motor cortex was noted (relative to controls). The change in iron was no longer significant.

### MRI findings in ALS patients

The volumes of the bilateral precentral and central motor cortex and the medulla oblongata were significantly lower in ALS patients than in controls (Tables 1 and 2). The upper or lower limb onset did not affect the cervical spinal cord parameters (Supplemental Fig. 1). The N-acetyl aspartate (NAA) peak was significant lower in the motor cortex.

**Table 1:** Comparison of baseline characteristics in patients vs. controls group.

	Patients (n = 40)	Controls (n = 21)	p-value
**Characteristics**			
*Age*	56.18 (10.50)	55.48 (13.43)	0.82
*Sex (men/women)*	33/7	15/6	0.34
**Bilateral pre-central and central (motor) cortex**			
*Volume*	0.56 (0.08)	0.67 (0.11)	**<0.0001**
*R2*	14.73 (1.54)	15.17 (1.69)	0.60
*NAA*	1.41 (0.19)	1.56 (0.18)	**0.005**
**Bilateral PLIC**			
*FA*	0.61 (0.05)	0.62 (0.09)	0.61
*MD* ^+^ *, median [IQR]*	7.21 [7.03 ‒ 7.53]	7.18 [6.75 ‒ 7.32]	0.32
**Medulla oblongata**			
*Volume*	0.20 (0.03)	0.24 (0.04)	**<0.0001**
*R2*	16.85 (2.54)	17.06 (2.44)	0.76
*FA*	0.33 (0.06)	0.35 (0.14)	0.56
*MD* ^+^ *, median [IQR]*	8.31 [7.87 ‒ 9.50]	9.02 [7.64 ‒ 13.64]	0.50
**Cervical spinal cord**			
*Volume, median [IQR]*	2437 [2117 ‒ 2736]	2689 [2354 ‒ 2999]	0.060
*R2, median [IQR]*	38.13 [35.01 ‒ 46.13]	37.60 [36.67 ‒ 40.44]	0.71

Data are expressed as the mean (SD), unless otherwise indicated. Volume (as a percentage of the intracranial volume and in mm^3^ for the spinal cord); R2* mean values (1/T2*) (in s^−1^) Abbreviations: PLIC: posterior limb of internal capsule; FA: fractional anisotropy; MD: mean diffusivity; NAA: N-acetyl aspartate; IQR: interquartile range; SD: standard deviation. ^**+**^In units × 10000.

**Table 2:** Change in the main functional outcomes (mean (SD) total ALSFRS-r score, bulbar ALSFRS-r score and SVC) over the course of the study.

Time (*), months	Total ALSFRS-r score	Bulbar ALSFRS-r score	SVC
0	37.94 (5.73)	10.77 (1.59)	101.81 (18.79)
3	33.81 (7.61)	10.10 (2.20)	89.37 (24.69)
6	32.74 (7.72)	10.19 (2.02)	87.27 (25.28)
9	32.76 (8.12)	10.71 (1.61)	79.70 (21.96)
12	32.00 (9.43)	10.33 (2.26)	82.53 (27.98)

Values are expressed as the mean (SD). Abbreviations: ALSFRS-r: Amyotrophic Lateral Sclerosis Rating Scale - Revised Version; SD: standard deviation. *Time since study inclusion.

### MRI findings associated with disease progression outcomes

High fractional anisotropy (FA) and low mean diffusivity (MD) at baseline were associated with a smaller decrease in the total Amyotrophic Lateral Sclerosis Rating Scale - Revised Version (ALSFRS-r) score between baseline and 12 months (Table 3). High MD at baseline was associated with a smaller decrease in the bulbar ALSFRS-r score between baseline and 12 months. A reduction in the volume of the cervical spinal cord between baseline and 3 months was associated with a significantly smaller decrease in slow vital capacity (SVC) and tended to be associated with survival (p = 0.07).

**Table 3:** Factors associated with disease progression outcomes.

	Survival		Total ALSFRS-r		Bulbar ALSFRS-r		Slow Vital Capacity	
	HR (95%CI)	p-value	Interaction coefficient ${{\rm{\beta }}}_{3}$ (SD) (#)	p-value^•^	Interaction coefficient ${{\rm{\beta }}}_{3}$ (SD) (#)	p-value^•^	Interaction coefficient ${{\rm{\beta }}}_{3}$ (SD) (#)	p-value
**At baseline**								
Bilateral pre-central and central (motor) cortex								
R2*	0.97 (0.76 to 1.22)	0.77	−0.02 (0.11)	0.84				
*Volume*	0.73 (0.46 to 1.17)	0.19	0.42 (1.79)	0.81				
*Naa*	0.96 (0.78 to 1.19)	0.73	0.29 (1.07)	0.79				
Bilateral PLIC								
*FA*	0.95 (0.89 to 1.01)	0.086	5.93 (2.88)	**0.045**				
*MD*^*+*^	2.32 (1.03 to 5.23)	0.043	−0.77 (0.36)	**0.036**				
Medulla oblongata								
R2*	1.02 (0.89 to 1.18)	0.78	0.005 (0.06)	0.93	0.01 (0.02)	0.61		
*Volume*	1.11 (0.97 to 1.26)	0.14	−5.55 (5.30)	0.30	−2.89 (1.86)	0.13		
*FA*	0.91 (0.49 to 1.71)	0.77	−0.54 (2.98)	0.86	0.03 (1.04)	0.98		
*MD*^*+*^	1.15 (0.98 to 1.34)	0.085	0.05 (0.09)	0.57	0.04 (0.03)	**0.021**		
Cervical spinal cord								
R2*	1.02 (0.97 to 1.07)	0.50	0.02 (0.02)	0.44			0.08 (0.07)	0.27
*Volume (¤)*	0.87 (0.44 to 1.71)	0.68	−0.27 (0.34)	0.43			−0.88 (1.15)	0.45
*Corrected Volume (Δ)*	0.93 (0.43 to 2.00)	0.85	−0.44 (0.37)	0.23			−1.29 (1.25)	0.31
**Variations between inclusion and 3 months**								
Bilateral pre-central and central (motor) cortex								
R2* difference (t3 − t0)	1.02 (0.71 to 1.46)	0.91	−0.10 (0.08)	0.20				
*Volume* difference (t0 − t3)	0.53 (0.16 to 1.72)	0.29	4.36 (2.60)	0.10				
*Naa* difference (t0 − t3)	1.13 (0.80 to 1.60)	0.49	0.33 (0.78)	0.68				
Bilateral PLIC								
*FA* difference (t3 − t0)	1.23 (0.22 to 6.75)	0.81	−0.01 (4.54)	0.99				
*MD* difference (t0 − t3) ^+^	2.15 (0.37 to 12.36)	0.39	−0.22 (0.62)	0.73				
Medulla oblongata								
R2* difference (t3 − t0)	0.96 (0.84 to 1.09)	0.51	−0.04 (0.04)	0.39	−0.02 (0.02)	0.46		
*Volume* difference (t0 − t3)	0.98 (0.72 to 1.33)	0.89	8.70 (8.02)	0.29	1.87 (4.72)	0.18		
*FA* difference (t3 − t0)	0.88 (0.29 to 2.64)	0.81	−1.62 (2.52)	0.53	0.04 (0.74)	0.96		
*MD* difference (t0 − t3) ^+^	1.08 (0.78 to 1.50)	0.63	0.03 (0.09)	0.69	0.01 (0.02)	0.53		
Cervical spinal cord								
R2* difference (t3-t0)	0.98 (0.88 to 1.09)	0.67	0.007 (0.02)	0.73			0.07 (0.08)	0.43
*Volume* difference (t0 − t3) (¤)	8.72 (0.80 to 95.07)	**0.07**	−0.85 (0.49)	0.095			−4.56 (1.48)	**0.005**
*Corrected Volume (t0* − *t3)(∆)*	1.19 (0.54 to 2.61)	**0.65**	−0.28 (0.24)	0.26			−3.37 (1.27)	**0.013**

Volume (as a percentage of the intracranial volume and in mm^3^ for spinal cord); R2* values (in s^−1^); diffusivity; magnetic resonance spectrometry

HR = hazard ratio calculated with Cox proportional hazards. Volume (as a percentage of the intracranial volume and in mm^3^ for the spinal cord); R2* mean values (1/T2*) (in s^−1^). The volume of the cervical spinal cord is determined from the middle of the 3rd vertebra to the middle of the 5^th^ vertebra. The corrected volume is the normalization of this volume by the cord length from C1 to C7.

Quantitative variables were expressed as the mean (standard deviation) if normally distributed or the median [interquartile range] if not. Categorical variables were expressed as the number (percentage). The normality of distribution was assessed using histograms and the Shapiro-Wilk test.

ALSFRS-r: Amyotrophic Lateral Sclerosis Rating Scale - Revised Version; CI: confidence interval; PLIC: posterior limb of internal capsule; FA: fractional anisotropy; MD: mean diffusivity; NAA: N-acetyl aspartate; SD = standard deviation; (#) Coefficient β_3 of a mixed model with the outcomes (global ALSFRS-r score or bulbar ALSFRS-r score or SVC) as the dependent variable. 〖At baseline: N = 40 patients.ALSFRS-r〗_ij = β_0 + β_1 t_ij + β_2 R2_i + β_3 R2_i*t_ij + γ_0i + γ_1i t_ij + ε_ij. tij = for any subject i, time in months elapsed from baseline to the current measure j of parameters. Variations between inclusion and 3 months: N = 26 patients. 〖ALSFRS-r〗_ij = β_0 + β_1 t_ij + β_2 R〖2(t3 − t0)〗_i + β_3 R〖2(t3 − t0)〗_i*t_ij + γ_0i + γ_1i t_ij + ε_ij. tij = for any subject i, time in months elapsed from 3 months to the current measure j of parameters. ^•^p-values for interactions tagged in bold are significant at p < 0.05. ^+^In units × 10000. (¤) in units/1000.

Data not shown: there were no differences for the Glx, mI and Cho peaks.

## Discussion

Changes in MRI findings between diagnosis and 3 months may be surrogate biomarkers for the progression of ALS respiratory disorders over 12 months, with an impact on survival and quality of life. The MRI’s predictive value for survival was almost statistically significant in a small group of patients, which suggests that the features of the cervical spinal cord have great potential as a biomarker^6^. Spinal cord imaging is difficult (due to frequent artefacts) but could be critical for ALS because it includes the respiratory centres.

Typical signs of ALS were observed at the time of diagnosis, including atrophy of the motor cortex and the medulla oblongata, and neuronal loss (i.e. a lower NAA peak), as reported elsewhere^2–4^.

The decrease in MD in the medulla oblongata over 3 months was associated with the progression of bulbar handicap. MD of the cortico-spinal tract typically increases over time in ALS, reflecting the degeneration of the fibers^2–4^. However, MD values could be higher when measured in complex tissue, such as medulla oblongata, where degeneration of one fiber bundle could cause the other fiber bundle to become more dominant^7^.

However, the severe alteration already present in the cortex and the variable progression seen in the medulla oblongata probably reduce the values of these structures as biomarkers.

As previously reported^8^, alterations (i.e. changes in FA and MD) in the white matter of the internal capsule following cortical atrophy appears to be better surrogate biomarkers for progression of the functional handicap.

Our experiments on a limb-onset model of SOD1 demonstrated that iron accumulation in the cervical spinal cord appeared at the time of symptom onset and thus preceded atrophy. Progressive iron accumulation (from the lumbar spinal cord to the cervical spinal cord) has been reported in an autopsy study^9^. The iron content was low when atrophy occurred, and this may explain why high iron levels were not observed with 3 T MRI in ALS patients at the time of diagnosis (i.e. 12 months after the symptom onset). A slight increase in iron content (according to 7 T MRI) has been observed in patients^10^. Iron accumulation may be a promising biomarker earlier in the disease (i.e. at symptom onset).

The confirmation of our present findings in a very large population might lead to (i) a change in the care of ALS patients, in order to predict the need for rehabilitation and non-invasive ventilation, and (ii) the development of more sensitive endpoints for pilot studies of neuroprotection.

## Methods

### Study participants

We included 41 consecutive limb-onset ALS patients (25 with upper limb onset and 15 with lower limb onset) and 21 healthy controls. We excluded one patient who was finally diagnosed with motor neuropathy; 40 patients were analysed. None of the participants displayed dementia. All patients underwent MRI at diagnosis (i.e. baseline) and again after 3 months. They were monitored over the long term to confirm the diagnosis and to determine the prognosis. All patients had possible or probable ALS, according to the El Escorial laboratory-supported criteria. The Amyotrophic Lateral Sclerosis Rating Scale - Revised Version (ALSFRS-r) score, the Medical Research Council scale for muscle strength and the slow vital capacity (SVC) were assessed within 2 days of the MRI session and then every three months (Tables 1 and 2). Patients with an advanced form of ALS (i.e. inapt for prolonged dorsal decubitus or with an SVC below 50% of the predicted value) were excluded. The exclusion criteria for FTD and dementia (Diagnostic and Statistical Manual of Mental Disorders-V) were applied. All participants provided their informed consent. The study was approved by the local independent ethics committee (CPP Nord Ouest IV, Lille, France). The study strictly followed the methods, the guidelines and the regulations described in the approved protocol (i.e. an ancillary study to the PULSE study: Protocol ID: 2013-A00969-36; ClinicalTrials.gov: NCT02360891).

### Human MRI

MRI data were acquired on a 3 Teslas (3 T) Philips Achieva scanner (Philips Healthcare, the Netherlands) using an 8-channel phased-array head coil. Sagittal 3D T1-weighted isometric millimetre images were acquired using a magnetization-prepared gradient echo sequence (echo time (TE)/repetition time (TR): 3.3/7.2; matrix size: 256 × 256 × 176 mm^3^) followed by a sagittal 3 Dimensions multi-echo gradient sequence (TE/TR first: 5 ms/56 ms; matrix: 256 × 256 × 167 mm^3^; voxel size: 1 mm^3^; 6 echoes; echo spacing: 9 ms) and an axial diffusion tensor imaging (DTI) sequence (TE/TR: 56 ms/12000 ms; matrix: 128 × 128 × 67 mm^3^; voxel size: 2 mm^3^; 32 directions) for the posterior limb of the internal capsule (PLIC) and the medulla. Magnetic resonance spectroscopy data were analyzed for the precentral gyri and medulla oblongata (respectively 20 × 32 × 20 mm^3^ and 13 × 15 × 20 mm^3^, TE/TR: 35 ms/2000 ms) (Supplemental Fig. 1). However, the medulla oblongata sequence (performed at the end of the acquisition) had too much artifacts to be analyzed. Spinal cord assessment included a sagittal T2 weighted fat-sat sequence (TE/TR: 80 ms/3000 ms; matrix: 512 × 512 × 14 mm^3^; voxel size: 0.5 × 0.5 × 3 mm^3^) and a sagittalT2* weighted sequence (TE/TR first: 5 ms/718 ms; matrix: 220 × 220 × 17 mm^3^; voxel size: 1 × 1 × 3 mm^3^; 6 echoes; echo spacing: 8 ms).

All the MRI data were anonymized before post-processing. The reader was blinded to the status of the subjects. Regions of interest were automatically segmented on T1-weighted images using Freesurfer software (http://surfer.nmr.mgh.harvard.edu/), except for the medulla oblongata and cervical spinal cord, which were manually segmented (cervical spinal cord: from the middle of the 3^rd^ vertebra to the middle of the 5^th^). The corrected volume is the normalization of this volume by the cord length from C1 to C7.

A T2*-weighted map was generated using Osirix® and registered in T1 space via rigid registration with SPM software (http://www.fil.ion.ucl.ac.uk/spm/software/spm12/). DTI images were processed using FSL software (https://fsl.fmrib.ox.ac.uk/fsl/) and the FA map was registered in T1 space via rigid transformation with SPM software. Spectroscopy signals were processed using the LC model (http://s-provencher.com/lcmodel.shtml) for the assessment of N-acetyl aspartate (NAA), glutamate and glutamine (Glx), myo-inositol (mI) and choline (Cho) peaks.

### SOD1^86R^ transgenic mice

All experiments were carried out in accordance with the “Principles of Laboratory Animal Care” and the current French and European Union legislative and regulatory framework (animal use authorization number: APAFIS#4269-2015112317225759). Protocols were approved by an Ethical Committee (Nord-Pas-de-Calais; CEEA75). The FVB-Tg(Sod1^G86R^ mice)M1Jwg/J mice (JAX Laboratories)^5^ were assessed at 70 days (i.e. an age at which these mice are devoid of motor symptoms but already present with weight loss), 85 days (when mice become symptomatic) and 100 days (when they are severely affected, before death at around 110 days)^5^. All analyses were blinded.

### MRI of mice

Seven-Tesla MRI was performed in a horizontal bore magnet (Bruker, Biospec, Ettlingen, Germany). To image the cervical spinal cord, a volume coil (internal diameter: 59 mm) was used for radio frequency emission and reception. During acquisition, animals were anesthetized with a mixture of air and isoflurane concentrate (1–2%, depending on the breathing). In the magnet, the mouse’s head was immobilized using a three point-fixation system (tooth-bar and ear-plugs, Bruker). The mouse’s body temperature was maintained at 37°c using warm water circulating inside the bed. The animal’s breathing was monitored with a sensor pillow placed on the abdomen. The geometry of the sagittal T2* sequence was set so as to obtain a median sagittal slice. The parameters were the following: TR = 1500 ms, and the echo time values were incremented by 5 ms and ranged from 4 ms to 60 ms. The geometric parameters were as follows: a square FOV of 20 mm, a 256-square matrix, and a slice thickness of 1 mm. The acquisition time for this sequence was 10 minutes. Regions of interest were first drawn manually on sagittal T2* images. The T2* value was obtained by applying a mono-exponential model to the data set described by the 12 echoes. The thickness of the cervical spinal cord was assessed on the first T2* echo images and then manually measured. Post-treatment images of T2* decay were generated using Paravision software (version 5.1, Bruker).

### Statistical analysis

Parameters at baseline were compared in patients vs. controls using Student’s t test or (for non-Gaussian variables) the Mann-Whitney U test.

We investigated the association between the MRI parameter and the change over time in each outcome, using linear mixed models with random coefficients. We considered the time, the baseline parameter, and its interaction with time as a fixed effect. The random effects were the intercept and the slope for time. The association between each MRI parameter at baseline and event-free survival was analysed using a Cox proportional hazard model. The survival time was defined as the time interval between the date of disease onset and the date of the ALS-related event (intermittent nasal positive pressure ventilation and/or death). Patients who did not experience the event were censored on the date of their last follow-up. Log linearity and proportional hazard assumptions were assessed using Martingales and Schoenfeld residuals.

MRI parameters (atrophy and R2*) in the SOD1 mice were analysed with ANCOVA adjusted on the baseline values (i.e. Day 70). All statistical tests were two-sided, with a threshold for statistical significance set to p < 0.05. All analyses were performed using SAS software (version 9.4, SAS Institute Inc., Cary, NC 27513, USA).

## Electronic supplementary material

Supplementary information
