# Supplementary material for: MRI of the cervical spinal cord predicts respiratory dysfunction in ALS
Source: Sci Rep. 2018 Jan 29;8:1828. doi: 10.1038/s41598-018-19938-2 (PMC5789036; doi:10.1038/s41598-018-19938-2)
Supplement: Supplementary file 1 — Supplementary information [file 41598_2018_19938_MOESM1_ESM.docx]

**MRI of the cervical spinal cord predicts respiratory dysfunction in ALS**

Grolez G MD,^1*^ M Kyheng,^2*^ Lopes R PhD,^3^ Moreau C MD, PhD,^1^ Timmerman K,^4^ Auger F PhD,^5^ Kuchcinski G MD,^3^ Duhamel A MD, PhD,^2^ Jissendi-Tchofo P MD, PhD,^3,6^ Besson P PhD,^3^ Laloux C PhD,^4^ Petrault M,^4^ Devedjian JC PhD,^4^ Thierry Pérez MD^7^, Pierre François Pradat MD, PhD,^8^ Defebvre L MD, PhD,^1^ Bordet R MD, PhD,^4^ Danel-Brunaud V MD,^1^ Devos D MD, PhD,^1,4^

**1** Service de Neurologie, Université de Lille, CHU de Lille, INSERM UMRS_1171, LICEND COEN Center, Lille, France

**2** Département de Biostastistiques, Université de Lille, CHU de Lille, Lille, France

**3** Service de Neuroradiologie, Université de Lille, CHU de Lille, INSERM UMRS_1171, LICEND COEN Center Lille, Lille, France

**4** Service de Pharmacologie, Médicale Université de Lille, CHU de Lille, INSERM UMRS_1171, LICEND COEN Center Lille, Lille, France

**5** Plateau d’imagerie préclinique, Université de Lille, CHU de Lille, Lille, France

**6** Department of Radiology, Neuroradiology section, Free University of Brussels, CHU Saint-Pierre, Brussels, Belgium

**7** Service de Pneumologie, Université de Lille, CHU de Lille, Lille, France

**8** Laboratoire d’Imagerie Biomédicale, CNRS, INSERM, Sorbonne Universités, UPMC Univ Paris 06 & Département de Neurologie, Centre référent SLA, APHP, Hôpital Pitié-Salpêtrière, Paris, France

**Supplemental-Figure 1: Manually segmented Region of interest**

A. Spinal cervical cord volume from the middle of the third cervical vertebrae to the middle of the fifth cervical vertebrae for R2* and volume analysis. Projection on a T2 sequence.

B. Motor cortex (red) medulla oblongata (blue), pons (green)

C Voxel acquired with magnetic resonance spectroscopy in left motor cortex (the same volume was acquired in right motor cortex).

**Supplemental Table 1: Comparisons of the parameters of the cervical spinal cord between upper limb and lower limb onset at baseline and the progression over 3 months.**

|  | Upper limb onset | | Lower limb onset | p-value |
| --- | --- | --- | --- | --- |
| At baseline (n=40) |  | |  |  |
| Cervical spinal cord | (n=25) | | (n=15) |  |
| *R2* | 36.8 (33.2 to 40.1) | | 42.1 (36.7 to 48.3) | 0.057 |
| *Volume* | 2419 (2119 to 2727) | | 2438 (2117 to 2698) | 0.81 |
| *Corrected volume* | 211 (184 to 249) | | 217 (201 to 242) | 0.39 |
| Variations between inclusion and 3 months (n=26) | | | |  |
| Cervical spinal cord | | (n=16) | (n=10) |  |
| *R2** difference (t3-t0) | | -0.96 (-4.22 to -0.54) | -0.08 (-7.33 to 0.50) | 0.65 |
| *Volume* difference (t0-t3) (¤) | | 35.0 (-302 to 217) | 73.5 (-137 to 241) | 0.62 |
| *Corrected volume difference (t0-t3)(𝛥)* | | 0.49 (-30.8 to 13.6) | 9.6 (-16.4 to 33.0) | 0.48 |
